# Supplementary material for: The Frenkel Line: a direct experimental evidence for the new thermodynamic boundary
Source: Sci Rep. 2015 Nov 5;5:15850. doi: 10.1038/srep15850 (PMC4633585; doi:10.1038/srep15850)
Supplement: Supplementary Information [file srep15850-s1.pdf]

Supplementary Materials for
The Frenkel Line: a direct experimental evidence for the new thermodynamic boundary

Dima Bolmatov[1],* Mikhail Zhernenkov[1],† Dmitry Zav'yalov[2],
Sergey N. Tkachev[3], Alessandro Cunsolo[1], and Yong Q. Cai[1]
[1] *Brookhaven National Laboratory, Upton, NY 11973, USA*
[2] *Volgograd State Technical University, Volgograd, 400005 Russia and*
[3] *Center for Advanced Radiation Sources, University of Chicago, Chicago, IL 60637, USA*

PRESSURE AND TEMPERATURE EFFECTS ON S(Q) PEAKS EVOLUTION

Here, we provide an additional evidence for the non-uniformity of the supercritical state, which is the subject of the paper. Previous studies showed that pair correlations depend strictly linearly on the pressure variations [1]. Therefore, we can remove the pressure dependence of the pair correlations and demonstrate the evolution of $S(q)$ peaks on temperature variations only. For the sake of simplicity we can represent the first $S(q)$ position as: 1st Peak position(nm^{-1}) = P(GPa)+20 when the temperature is fixed. This dependence is derived from $S(q)$ peak positions reported in Figure 2 of the Ref. [1]. In general, the $S(q)$ peak position on the pressure-temperature diagram depends both on the temperature and the pressure. In the P-T range used in our experiment, we can simply approximate such a dependence using the following equation

$$Peak\ position = P + 20 + F(T) \tag{1}$$

Please note, that we added the dependence on temperature $F(T)$, for which there is no prior work available and we assume it is arbitrary. In order to obtain the pure dependence on the temperature T, we simply need to subtract the dependence of pressure P which is linear, as was shown previously [1]. Using the simple equation

$$F(T) = Peak\ position\ - P(GPa) - 20 \tag{2}$$

Both the first (see Fig. 1) and the second (see Fig. 2) $S(q)$ peaks are shown in arbitrary units according to Eq. 2 (we neglected the constant "20" which is just an offset). It is clearly seen that both kinks are well preserved (same as

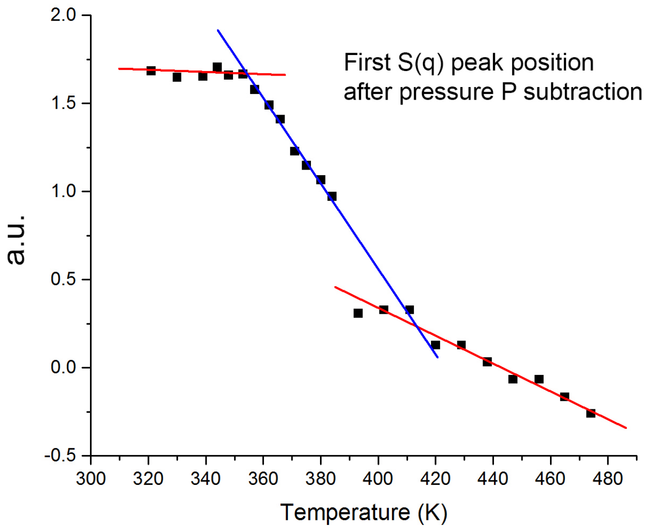

FIG. 1: **Experimental evolution of the 1st $S(q)$ peak across the Frenkel line**. The first $S(q)$ peak is shown in arbitrary units without pressure contributions. The emergence of two kinks proves the existence of the new thermodynamic boundary in the supercritical state.

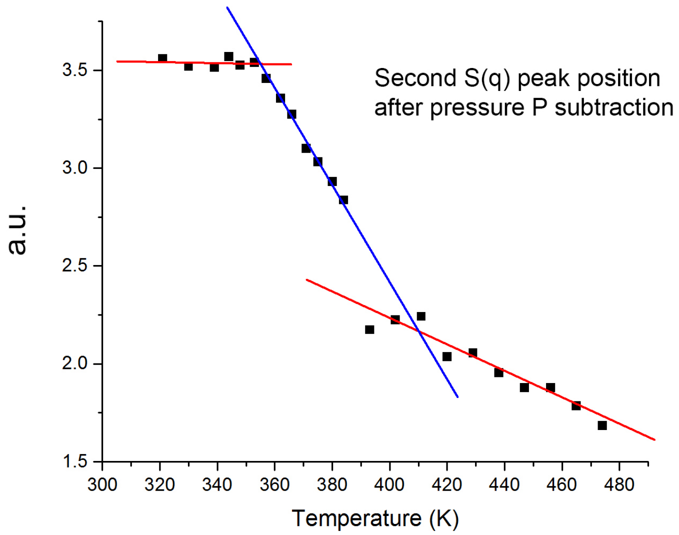

FIG. 2: **Experimental evolution of the 2nd $S(q)$ peak across the Frenkel line**. The second $S(q)$ peak is shown in arbitrary units without pressure contributions. The appearance of two kinks is the manifestation of the Frenkel line.

in the main text, see Figs. 2-3) which proves again the validity of the results in the main text. The "zigzag" behavior of the $S(q)$ peaks (non-uniformity) is the manifestation of the Frenkel line.

* Electronic address: `d.bolmatov@gmail.com,bolmatov@bnl.gov`
† Electronic address: `zherne@bnl.gov`

[1] Santoro, M. & Gorelli, F. A. Structural changes in supercritical fluids at high pressures. Phys. Rev. B **77**, 212103 (2008).
